# ISBDD Model for Classification of Hyperspectral Remote Sensing Imagery

**DOI:** 10.3390/s18030780

**Published:** 2018-03-05

**Authors:** Na Li, Zhaopeng Xu, Huijie Zhao, Xinchen Huang, Zhenhong Li, Jane Drummond, Daming Wang

**Affiliations:** 1 School of Instrumentation Science and Opto-Electronics Engineering, Beihang University, Beijing 100191, China; ZY1517323@buaa.edu.cn (Z.X.); gogo422@buaa.edu.cn (X.H.); 2 School of Engineering, Newcastle University, Newcastle upon Tyne NE1 7RU, UK; zhenhong.li@newcastle.ac.uk; 3 School of Geographical and Earth Sciences, University of Glasgow, Glasgow G12 8QQ, UK; jane_drummond_29@talktalk.net; 4 China Geological Survey, Beijing 100037, China; wangdaming@mail.cgs.gov.cn

**Keywords:** hyperspectral, classification, training samples with interference, multi-instance learning, diverse density

## Abstract

The diverse density (DD) algorithm was proposed to handle the problem of low classification accuracy when training samples contain interference such as mixed pixels. The DD algorithm can learn a feature vector from training bags, which comprise instances (pixels). However, the feature vector learned by the DD algorithm cannot always effectively represent one type of ground cover. To handle this problem, an instance space-based diverse density (ISBDD) model that employs a novel training strategy is proposed in this paper. In the ISBDD model, DD values of each pixel are computed instead of learning a feature vector, and as a result, the pixel can be classified according to its DD values. Airborne hyperspectral data collected by the Airborne Visible/Infrared Imaging Spectrometer (AVIRIS) sensor and the Push-broom Hyperspectral Imager (PHI) are applied to evaluate the performance of the proposed model. Results show that the overall classification accuracy of ISBDD model on the AVIRIS and PHI images is up to 97.65% and 89.02%, respectively, while the kappa coefficient is up to 0.97 and 0.88, respectively.

## 1. Introduction

Hyperspectral remote sensing is widely used in water resources, air quality monitoring, and agricultural and soil properties research because of its high spectral resolution [1,2,3,4]. The high classification accuracy of hyperspectral images is the precondition of its effective application. However, the classification of hyperspectral images encounters many problems, such as the “curse of dimensionality”, nonlinear data and spatial heterogeneity. Many techniques have been proposed to handle these problems. The feature mining technique is proposed to reduce the dimension to decrease the impact of the “curse of dimensionality” by mining the dimension of the hyperspectral image [5]; the kernel function transformation technique works well when dealing with nonlinear data [6]; and spectral-spatial clustering considered both the spectral information and spatial correlativity to reduce the impact of the spatial heterogeneity problem [7]. Additionally, research shows that the classification accuracy is reduced if training samples contain interference such as mixed pixels in supervised classification [8,9]. 

In the classification of drug protein molecules, Dietterich et al. [10] proposed a multi-instance learning (MIL) method to handle the problem of training samples with interference. Later, the DD algorithm was proposed by Maron and Lozano-Perez to learn a simple description of a person from a series of images containing that person [11], and became a classical algorithm for the MIL method. A LiMu et al. applied the DD algorithm to classifying the high-resolution remotely sensed image and gained better result than the support vector machine classifier [12]. For image retrieval, Wada et al. combined the K nearest neighbor algorithm and the DD algorithm for content-based image retrieval. Their work improved the accuracy of image retrieval and retrieval efficiency because the new method reduces the impact of irrelevant information, which is regard as interference factors in the image [13]. In these research fields, the DD algorithm performed effectively in dealing with the interference problem. Bolton et al. emphasized the reasonable application of the DD algorithm to hyperspectral image for image typically sensed from a distance to allow for image formation errors and spectral mixing [14,15,16]. 

In the DD algorithm, only one feature vector is learned from the training bags of a type of ground cover to represent this type of ground cover. Then the DD classifier will classify each pixel in the hyperspectral image according the feature vectors which can represent different types of ground cover. However, only use one feature vector cannot represent one type of ground precisely, because the spectral characteristics of different pixels for one type of ground cover may have some interference in certain spectral bands. The interference may make different pixels of one type of ground cover slightly different in terms of their spectral characteristics [8]. In the present study, the instance space-based diverse density (ISBDD) model, which employs a novel training strategy, is proposed to deal with this problem. In the ISBDD model, the DD values of each pixel are computed according to the training bags of different ground covers, and the pixels are classified according to their DD values. Thus, the pixels in hyperspectral images will not be classified according to the learned feature vectors of different types of ground cover; instead, they will be classified directly according to the training bags of different types of ground cover. The proposed ISBDD model greatly increases the classification accuracy of hyperspectral image because of the new training strategy.

## 2. Materials and Methods 

### 2.1. DD Algorithm

The DD algorithm was proposed to handle the problem of training samples with interference [11]. The most basic unit in the DD algorithm is called an instance, which is actually a pixel in a hyperspectral image. The two types of instances are positive and negative [17]. If an instance belongs to a certain type of ground cover, then it is called a positive instance for this type of ground cover, otherwise it is called a negative instance. An area in the image that contains several pixels (instances) is called a training bag. The two types of training bags are as follows: (i) a positive training bag, which contains at least one positive instance; and (ii) a negative training bag, which contains only negative instances [18]. The purpose of the DD algorithm is to learn a feature vector from the feature space to represent the training bags. The learning strategy of a feature vector is that the feature vector must be similar to positive bags and not to negative bags [19]. The similarity can be measured by a probability value computed from the distance between the feature vector and the training bags. The probability value is known as the DD value.

The mathematical description of the DD algorithm is as follows. 

Assume that $B_{i}^{+}$ is the i-th positive bag and $B_{ij}^{+}$ is the j-th instance in the i-th positive bag, while $B_{ijk}^{+}$ is the k-th attribute of the j-th instance. Similarly, $B_{i}^{-}$ is the i-th negative bag and $B_{ij}^{-}$ is the j-th instance in the i-th negative bag, and $B_{ijk}^{-}$ is the k-th attribute of the j-th instance. A multi-dimensional vector in the feature space is marked x, and the maximum DD value is represented by $t_{\max}$. $\mathrm{Pr}(x=t|B_{1}^{+},B_{2}^{+},......,B_{n}^{+},B_{1}^{-},......,B_{n}^{-})$ is the probability of an instance that belongs to a certain type of ground cover. The learning purpose is to dig $t_{\max}$ out and learn a representative feature vector through maximizing $\mathrm{Pr}(x=t|B_{1}^{+},B_{2}^{+},......,B_{n}^{+},B_{1}^{-},......,B_{n}^{-})$. Supposing each instance is subject to independent distribution, according to Bayesian theory [20]:

(1) $$t_{\max}=\max\prod_{i}\mathrm{Pr}(x=t|B_{i}^{+})\prod_{i}\mathrm{Pr}(x=t|B_{i}^{-})$$

Maron and Lozano-Perez used the noisy-or model to embody Equation (1):

(2) $$\mathrm{Pr}(x=t|B_{i}^{+})=1-\prod_{j}\left(1-\mathrm{Pr}(x=t|B_{ij}^{+})\right)$$

(3) $$\mathrm{Pr}(x=t|B_{i}^{-})=\prod_{j}\left(1-\mathrm{Pr}(x=t|B_{ij}^{-})\right)$$

The similarity between x and an instance in the training bag $B_{ij}$ is defined in the form of Equation (4).

(4) $$\mathrm{Pr}(x=t|B_{ij})=\exp(-||B_{ij}-x|{|}^{2})$$

### 2.2. Classification of Hyperspectral Remotely Sensed Image Based on the DD Algorithm

**Figure 1:**
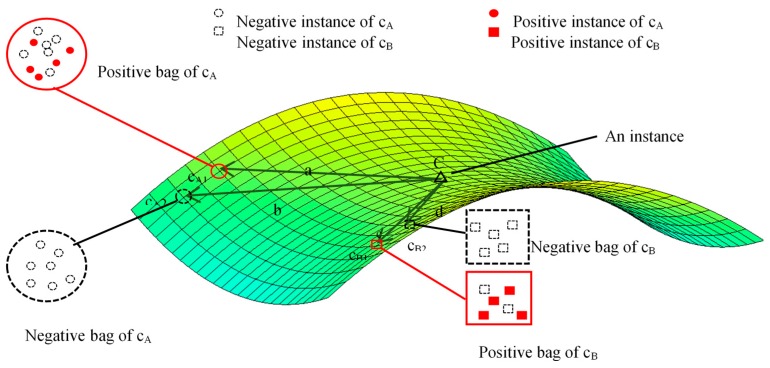
Training process of the DD algorithm.

The interference in hyperspectral remotely sensed images mainly contains mixed pixels, noise, large dispersion degree in one class and so on. In the imaging process of hyperspectral image sensors, mixed pixels are common in the resulting hyperspectral image because of the limitation of spatial resolution of sensors, especially at the border of two types of ground cover. Mixed pixels that contain the spectral characteristics of several types of ground cover may reduce the classification accuracy of the hyperspectral image. Also, noise such as measurement uncertainty also exists in hyperspectral images. In general, the supervised classification task involves training and classification processes [21]. In traditional supervised classification, the training samples with interference selected from the hyperspectral image is regard as a pure training sample. This will reduce the classification accuracy of hyperspectral image. In the DD algorithm, the influence of training samples with interference is considered in the training process. The training process of the DD algorithm is described in Figure 1. Assume that the curved surface shown in Figure 1 represents a feature space, and C_A_, C_B_ are two types of ground covers. The positive bag of C_A_ is represented by C_A1_, and the negative bag of C_A_ is represented by C_A2_. Similarly, C_B1_ and C_B2_ are the positive and negative bags of C_B_, respectively. C is an instance, which is a potential feature vector of a certain type of ground cover. Now we want to judge whether instance C is more likely to represent C_A_ or C_B_. In this case, the distance between C and C_A1_ is the same as the distance between C and C_B1_, and the distance of C and C_A2_ is greater than that of C and C_B2_. Therefore, C is more likely to represent C_A._

Generally, $c_{1},c_{2},......,c_{k}$ are K types of ground covers and $c_{ij}$ is the j-th bag of the i-th ground cover. The spectral dimension of the hyperspectral image is represented by n. $c_{i1},c_{i2},......,c_{im}$ are m bags of $c_{i}$. In the training process, a feature vector is learned according to the positive bags and negative bags of the i-th type of ground cover. In the hyperspectral image, the negative bag of one type of ground cover is the positive bag of the other type of ground cover. The goal of the training process is to learn a feature vector, which can represent the training bags of a certain type of ground cover. The process can be described by Formula (5), as follows:

(5) $$f(c_{i1},c_{i2},......,c_{im})=f_{i}$$

where $f_{i}$ is the learned feature vector of $c_{i}$, and the f function represents the training process. In the classification process, a pixel in the hyperspectral image can be classified according to the feature vector.

The classification result of the DD model is determined by the feature vector, which was learned in the training process. However, only use one feature vector alone cannot represent all the pixels of a type of ground cover, and this will influence the performance of the DD model. In order to handle this problem, the ISBDD model is proposed.

### 2.3. ISBDD Model for Classification of Hyperspectral Remotely Sensed Image

The ISBDD model employs a new training strategy to carry out the training process. In the DD algorithm, only one feature vector is used to represent the training bags. However, only one feature vector alone cannot represent one type of ground cover well. In the new training strategy, we do not learn a feature vector from the training bags. Instead, we classify the unknown pixels according to DD values of the unknown pixel, which is computed from the training bags of all the types of ground cover.

Suppose that k types of ground cover and N pixels exist in a hyperspectral image. The vector of an input instance (pixel) is represented as $x_{i}\in R^{n}(i\leq N)$, and the output label is represented as $y\in \{c_{1},c_{2},......,c_{k}\}$, where $c_{j}$ represents the j-th type of ground ground cover. $v_{i1},v_{i2},......,v_{ik}$ are DD values of $x_{i}$ computed according to training bags of these k types of ground covers , where $v_{im}$ is the DD value of pixel $x_{i}$ computed from the training bags of the m-th type of ground cover. The formula to compute $v_{im}$ is as follows:

(6) $$v_{im}=\prod_{l}\mathrm{Pr}(x_{i}=t|B_{q}^{+})\prod_{l}\mathrm{Pr}(x_{i}=t|B_{q}^{-})$$

where l is the number of training bags for the m-th type of ground cover. 

The formula is also embodied through the noisy-or model as follows:

(7) $$\mathrm{Pr}(x_{i}=t|B_{q}^{+})=1-\prod_{j}\left(1-\mathrm{Pr}(x_{i}=t|B_{qj}^{+})\right)$$

(8) $$\mathrm{Pr}(x_{i}=t|B_{q}^{-})=\prod_{j}\left(1-\mathrm{Pr}(x_{i}=t|B_{qj}^{-})\right)$$

After the training process, each pixel obtains k DD values, and the label of pixel $x_{i}$ is gained using Formula (9), as follows:

(9) $$y=\arg_{ci}\max\{v_{i1},v_{i2},......,v_{ik}\}$$

**Figure 2:**
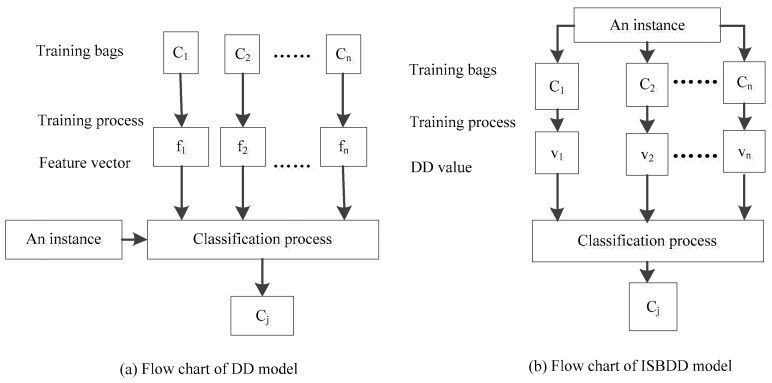
Flow chart of the DD and ISBDD models.

The processes of the DD model and the ISBDD model are compared in Figure 2. In the training process of the DD model, the feature vector $f_{i}$ is learned, whereas in the ISBDD model, learning a feature vector is not required to represent the training bags. In the new model, we directly compute according to the training bags, and all the information provided by the training bags is utilized in the training process to deal with the noisy training samples. For example, a hyperspectral image that contains two types of ground cover needs to be classified. In the ISBDD model, firstly, the training samples of the two types of ground cover are selected according to the ground truth image or the ground survey image. Then, each pixel in the hyperspectral image which need to be classified is looped through, and the diverse density values of the pixel and the training sample bags of the two types of ground cover are computed according to Formula (6). Finally, the pixel is classified as a certain type of ground cover according to its diverse density values.

### 2.4. Experiment Description

The experiment for the classification of hyperspectral images was implemented on a PHI image and an AVIRIS image. The airborne hyperspectral image collected by PHI covers the Fanglu Tea plantation area in Jiangsu province of China, which is situated at (31°40′39″ N, 119°22′53″ E). We used an image with a size of 200 × 150 pixels and 65 spectral channels in the experiment. The image was collected on October 2002. The area where the image covers contains 7 types of ground cover, namely paddy, caraway, wild-grass, pachyrhizus, tea, bamboo, and water. The spatial resolution of the PHI image is 2 m. The AVIRIS image named Indian Pines covers the agricultural demonstration zone in Northwest Indiana of the US. We use an image with a size of 145 × 145 pixels and 224 spectral channels. It was collected on June 1992. The area where the image covers contains 16 types of ground cover, namely, alfalfa, corn-min (corn seeding), corn, grass/trees, grass/pasture, grass/pasture-moved (trimmed grass/pasture), hay-windrowed, oats, soybeans-notill (no-till soybeans), soybeans-min, soybean-clean (cleaned soybeans), wheat, woods, bldg-grass-tree-drives, stone-steel towers and corn-notill. The spatial resolution of the AVIRIS image was 20 m. We used the AVIRIS image to test the feasibility of ISBDD and then used the PHI image to test the applicability of ISBDD.

**Figure 3:**
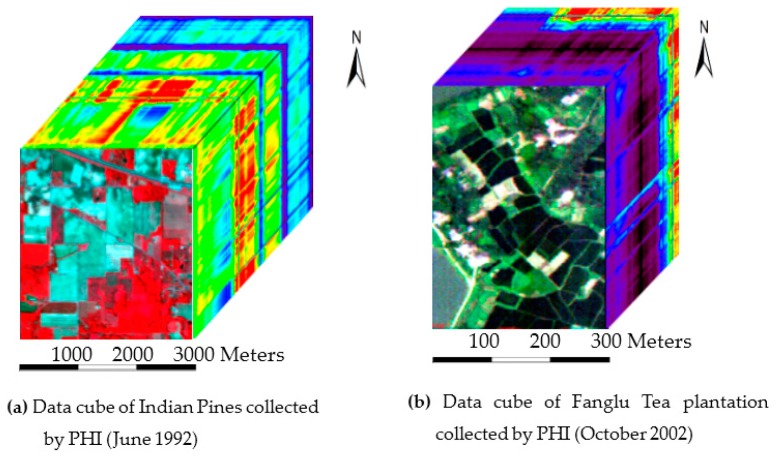
Data cube of images collected by AVIRIS and PHI.

**Figure 4:**
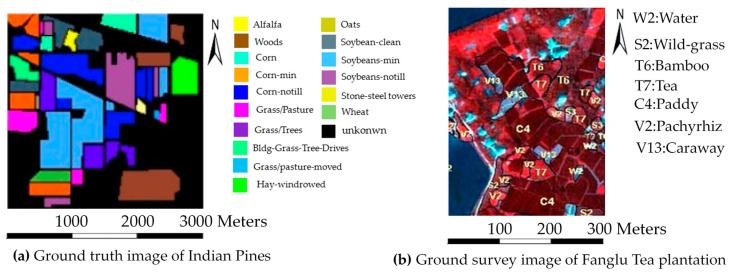
Distribution of ground cover in the two images collected by AVIRIS and PHI.

We call the hyperspectral image a data cube because it consists of two dimensions in the spatial dimension and one dimension in the spectral dimension. Figure 3 shows the data cube of the two images collected by AVIRIS and PHI, and Figure 4 shows the distribution of ground cover in the two images. Figure 4a is the ground truth image of the Indian Pines and Figure 4b is the ground survey image of the Fanglu Tea plantation. The two images can describe the distribution of ground cover. In Figure 4a, different color represents different types of ground cover, and the black color in the ground truth image of Indian Pines represent area where the ground cover is unknown.

**Figure 5:**
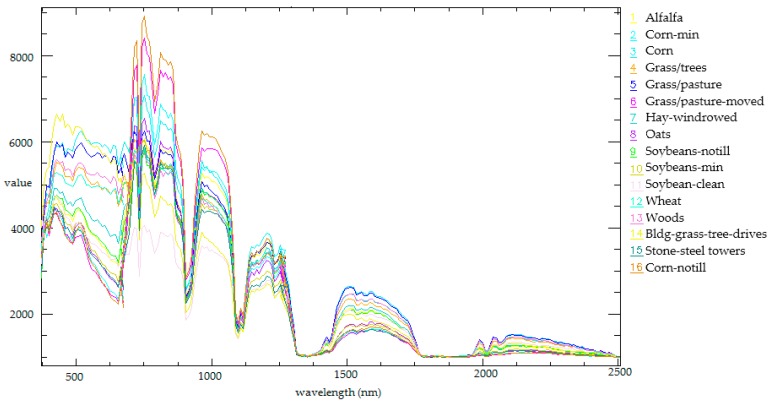
Spectral characteristic of 16 types of ground covers covered in the Indian Pines.

The spectral characteristic of seven types of ground cover in the Indian Pines are shown in Figure 5. The training and testing samples are selected from the image, and their specific information is shown in Table 1. In this case, the training samples without interference for a certain type of ground cover indicate that the labels of all pixels in the training samples are consistent with the label of a certain type of ground over. The training samples with interference for a certain class indicate that the training samples contain pixels whose labels are inconsistent with the label of a certain type of ground cover. In the stage of sample selection, we choose the pixels which has the inconsistent label as the interference for a type of ground cover. We choose pixels with interference and pixels without interference based on the ground truth image of the Indian Pines and the ground survey image of the Fanglu Tea plantation.

**Figure 6:**
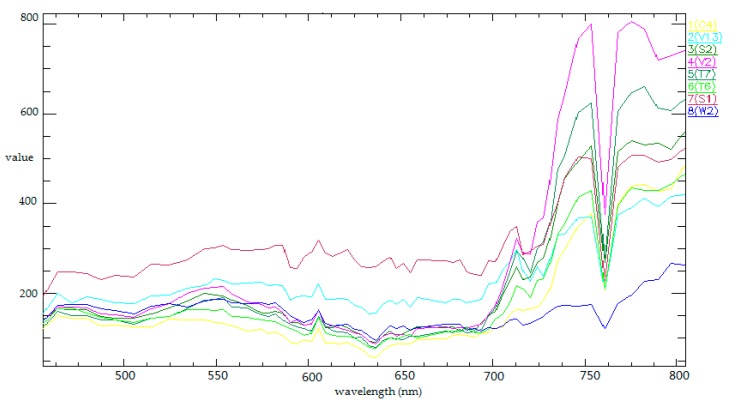
Spectral characteristic of seven types of ground cover covered in the Fanglu Tea plantation.

The spectral characteristic of the seven types of ground cover in Fanglu Tea plantation area are shown in Figure 6. The training samples and testing samples are selected from the hyperspectral image, and the specific information is shown in Table 2.

The maximum likelihood (MLC) algorithm is a classical classification algorithm for remotely sensed images, and the support vector machine (SVM) method has been a popular and effective classification algorithm in recent years [22]. We compare the classifier based on MLC and SVM algorithm with the classifier based on ISBDD algorithm. To fully verify the ability of the ISBDD classifier, the DD classifier is utilized. The kernel function of the SVM classifier is the radial basis function. We select the tolerant penalty parameter of the SVM classifier. To verify the performance of the ISBDD model, pixels with interference, which do not belong to a certain type of ground cover, are introduced to the training samples. Thereby, we use the training samples with interference to train the ISBDD and DD classifiers. The training samples without interference are a subset of the training samples. The MLC and SVM classifiers are applied to the hyperspectral image using the training samples without interference and the training samples with interference. 

The training samples of the two hyperspectral images are divided into five subsets, and the number of training samples in each subset is equal. Five rounds of experiment are conducted, and the final classification result is the average of the five results. The design of a randomized block experiment can reduce the impact of random factors and avoid the over-fitting of the results. The results are evaluated based on the overall accuracy, kappa coefficient and accuracies of each class. 

## 3. Results

### 3.1. AVIRIS Image

**Figure 7:**
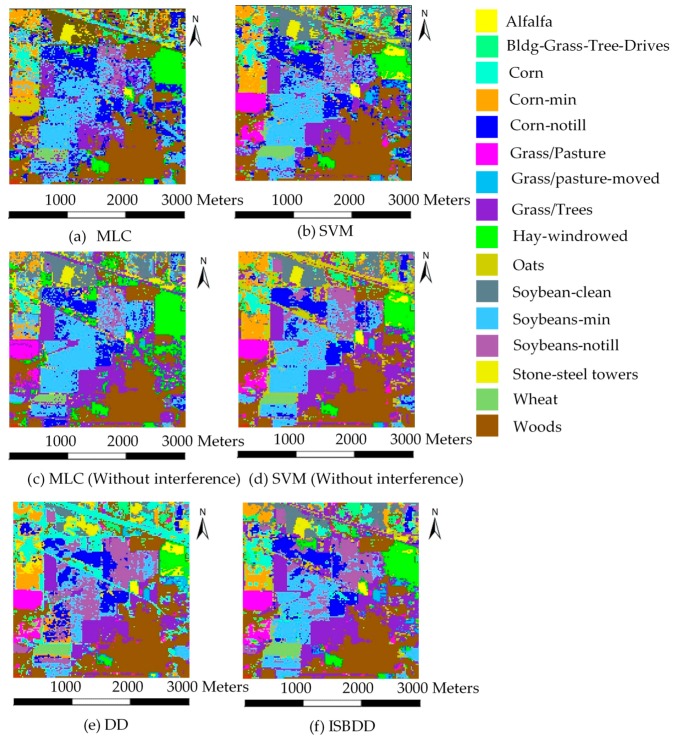
Classified images of the Indian Pines.

We use the AVIRIS image to test the feasibility of ISBDD. Figure 7 shows the classified images of the Indian Pines. Figure 7a–f shows the images classified by the MLC classifiers, SVM classifiers, DD and ISBDD classifiers.

The classification performance can be measured by the classification accuracy of each type of ground cover, the overall accuracy, and the kappa coefficient. The classification accuracy of each type of ground cover can be used to evaluate the classification accuracy of each ground cover, and the overall accuracy can be used to evaluate the performance of a classifier on the entire image. The kappa coefficient can be used to evaluate the consistency of the classification results and the actual distribution. Table 3 shows the average classification accuracy comparison for the purpose of evaluating the performance of the four classifiers. The maximum accuracy is highlighted in bold.

Figure 7 and Table 3 show that the DD and ISBDD classifiers perform better than the MLC and SVM classifiers when the training samples contain interference. The ISBDD classifier performs best in the classification accuracy of each type of ground cover because it has the highest classification accuracy in 10 of the 16 types of ground cover. The capacity of the DD and ISBDD classifiers to deal with the interference problem benefits from their MIL framework. The ISBDD classifier performs better than the MLC and SVM classifiers even if its training samples contain interference, whereas the training samples of the MLC and SVM classifiers do not contain interference. However, the DD classifier is not effective in this situation because the ISBDD classifier employs a novel training strategy in the training process. The result proves that the use of ISBDD is feasible for handling the interference problem. 

### 3.2. PHI Image

**Figure 8:**
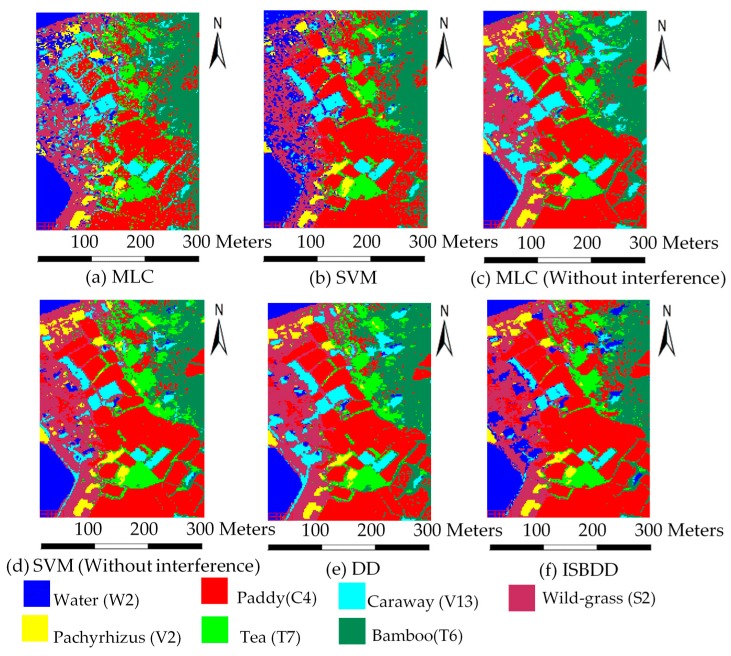
Classified images of the Fanglu Tea plantation.

We use the AVIRIS image to test the applicability of ISBDD. The classified images of the Fanglu tea plantation are shown in Figure 8. Figure 8a–f shows the images classified by the four classifiers.

Table 4 shows the average classification accuracy comparison for the purpose of evaluating the performance of the four classifiers. The maximum precision is highlighted in bold.

Figure 8 and Table 4 show that the classification accuracy of MLC and SVM is the lowest when the training samples contain interference. However, when the training samples are pure, the classification accuracy of these classifiers is high, especially for the SVM. The ISBDD classifier performs best in the classification accuracy of each type of ground cover because it has the highest classification accuracy in three of the seven types of ground cover. The overall accuracy of ISBDD is also the highest, while the classification accuracy of DD is lower than that of the SVM (without interference). The results demonstrate the effectiveness of ISBDD.

The classification results of the two hyperspectral images demonstrate the feasibility and applicability of the ISBDD model when the training samples contain interference. When the training samples are not contain interference, the MLC classifier and the SVM classifier can achieve satisfactory classification accuracy. However, when the training samples contain interference, the advantage of the ISBDD model is apparent. 

## 4. Discussion

### 4.1. Influence of Interference Intensity 

**Figure 9:**
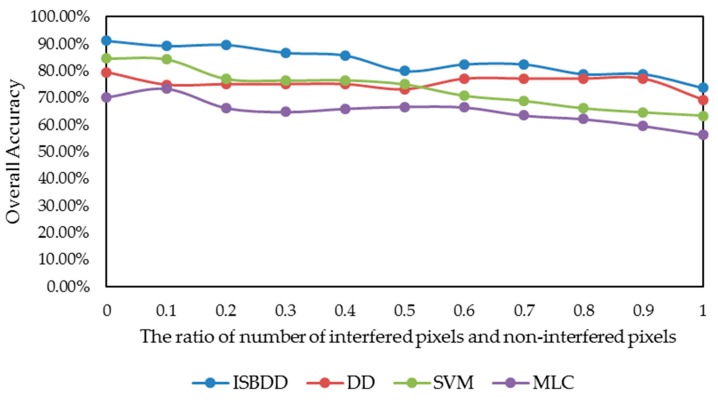
The impact of intensity of interference on classification accuracy for the Indian Pines.

The purpose of the DD and ISBDD model is to deal with problem of interference, wherein the proportion of pixels with interference can modulate the performance of the classifiers. Figure 9 shows the impact of the proportion of pixels with interference in the training samples on classification accuracies using the four classifiers for the Indian Pines image. The *x*-axis is the ratio of pixels with interference to pixels without interference in the training samples, indicating the intensity of interference. The *y*-axis shows the overall accuracy.

Figure 9 shows that the overall accuracy of the ISBDD classifier is higher than that of the other classifiers. In cases where the ratio is larger than 0.5, the overall accuracy of the MLC and SVM classifier declines sharply, whereas the overall accuracy of the DD and ISBDD classifier declines slowly. However, the decline range of overall accuracy from 0 to 0.9 of the ISBDD classifier is almost the same as that of the MLC classifier, and these results demonstrate the advantage of ISBDD in dealing with the interference problem. 

**Figure 10:**
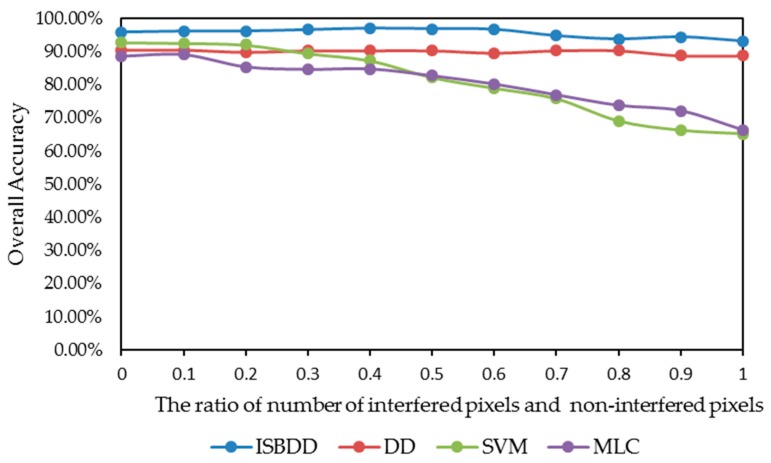
The impact of intensity of interference on classification accuracy for the Fanglu tea plantation.

Figure 10 illustrates the impact of the intensity of pixels with interference in training samples on classification accuracy when the four classifiers are used for the Indian Pines image. The *x*-axis shows the ratio of the pixels with interference to pixels without interference in the training samples. The *y*-axis shows the overall accuracy.

Figure 10 shows that the overall accuracy of the ISBDD classifier is higher than that of the other classifiers. The DD classifier does not perform better than the SVM or the MLC classifier when the ratio of pixels with interference and pixels without interference is smaller than 0.2. However, when the ratio is larger than 0.4, the overall accuracy of the MLC and SVM classifier declines sharply, whereas the overall accuracy of the DD and ISBDD classifier declines slowly. The result shows that the MLC classifier and the SVM classifier is more sensitive to interference than the ISBDD classifier.

### 4.2. Application Prospects and Future Work 

An increasing number of satellites equipped with hyperspectral camera are being launched for the purpose of crop survey, military target detection, and so on. The research and application of pattern recognition, such as face detection, is also gaining popularity. All these fields are concerned with supervised classification, in which the interference problem is inevitable when selecting the training samples. The ISBDD can be used in supervised classification to handle the problem of training samples with interference. The training strategy of ISBDD provides the model the powerful capability to complete the classification task though the training samples that contain interference.

However, the high accuracy cost of the ISBDD classifier is that its computational complexity is higher than that of the DD classifier. Suppose that w is the width of a hyperspectral image, and h is the height of a hyperspectral image, then the w×h pixels exist completely in the hyperspectral image. Assuming that a total of N pixels exist in the positive bags and the image contains c types of ground cover, then in the training process, most computing resources are used to determine the DD value. Thus, the ratio of the computational complexity of the ISBDD classifier to that of the DD classifier can be described as follows:

(10) $$R=\frac{w\times h\times c}{N}$$

In general, the numerator of Formula (10) is greater than the denominator. Thus, the computational complexity of the ISBDD classifier is considerably higher. The efficiency of the ISBDD classifier should be improved because, in many situations, efficiency is crucial. One of the solutions for improving efficiency is to utilize the multi-thread technique and parallel computing, given that the computing process for each pixel in the ISBDD classifier is independent.

## 5. Conclusions 

The ISBDD model, which employs a novel training strategy, is proposed in this paper. The major contribution of this work is in exploring a model with high classification accuracy to handle the problem of training samples with interference in the classification of hyperspectral image. In the proposed model, instead of learning a feature vector to represent the training bags, pixels are classified directly according to training bags. The ISBDD model is compared with several classical hyperspectral image classification models, including the diverse density model. Results demonstrated that the classifier based on the ISBDD model performs better than that based on other algorithms. Specifically, the overall accuracy of the ISBDD classifier is approximately 8% higher than that of the DD classifier. The overall accuracy of the proposed classifier is much higher than the SVM and MLC classifiers when the training samples contain interference. The results show that the ISBDD classifier can handle the interference problem effectively. In addition, it can be used in classification scenarios, especially when an accurate classification is highly required. However, the computational complexity of the ISBDD model is higher than that of the DD classifier because several DD values are computed for each pixel. Thus, the multi-thread technique and parallel computing can be utilized to increase the efficiency.

## Figures and Tables

**Table 1:** Number of training samples and testing samples for the Indian Pines.

Class ID	Class Name	Training Samples		Testing Samples
		Without Interference	With Interference	
1	Alfalfa	25	32	18
2	Corn-min	28	35	81
3	Corn	30	38	54
4	Grass/trees	31	40	161
5	Grass/pasture	25	30	80
6	Grass/pasture-moved	20	25	12
7	Hay-windrowed	50	65	64
8	Oats	20	25	10
9	Soybeans-notill	29	37	155
10	Soybeans-min	39	53	184
11	Soybean-clean	38	48	75
12	Wheat	30	40	60
13	Woods	38	48	156
14	Bldg-grass-tree-drives	30	25	60
15	Stone-steel towers	35	45	16
16	Corn-notill	30	38	178

**Table 2:** Number of training samples and testing samples for the Fanglu Tea plantation.

Class ID	Class Name	Training Samples		Testing Samples
		Without Interference	With Interference	
1(W2)	Water	92	120	954
2(C4)	Paddy	195	255	976
3(V13)	Caraway	105	138	295
4(S2)	Wild-grass	105	135	382
5(V2)	Pachyrhizus	66	82	211
6(T7)	Tea	105	135	411
7(T6)	Bamboo	135	180	443

**Table 3:** Average classification accuracy comparison of the four classifiers.

Method	MLC	SVM	MLC (Without Interference)	SVM (Without Interference)	DD	ISBDD
Alfalfa (%)	85.56	**100.0**	81.11	**100.00**	**100.0**	**100.0**
Corn-min (%)	56.22	67.55	66.71	**96.22**	77.20	91.19
Corn (%)	97.14	**98.57**	97.14	**98.57**	24.29	75.71
Grass/trees (%)	60.40	75.30	79.06	**99.73**	91.41	95.70
Grass/pasture (%)	81.00	**100.0**	87.17	**100.00**	100.0	**100.0**
Grass/pasture-moved (%)	63.33	**100.0**	11.67	**100.00**	**100.0**	**100.0**
Hay-windrowed (%)	99.87	90.67	**99.87**	89.87	77.74	89.87
Oats (%)	24.00	92.00	8.00	**100.00**	88.00	**100.0**
Soybeans-notill (%)	32.39	57.32	28.31	56.34	**77.32**	76.62
Soybeans-min (%)	78.16	88.78	88.57	85.30	77.96	**94.90**
Soybean-clean (%)	**100.0**	**100.0**	**100.00**	**100.00**	93.21	**100.0**
Wheat (%)	95.67	**100.0**	97.33	**100.00**	100.0	**100.0**
Woods (%)	98.10	99.05	98.33	99.29	93.33	**100.0**
Bldg-grass-tree-drives (%)	11.00	34.33	7.33	39.67	50.67	**59.00**
Stone-steel towers (%)	**100.0**	**100.0**	**100.00**	98.40	29.60	99.20
Corn-notill (%)	40.95	35.81	18.86	47.43	60.57	**65.52**
Overall accuracy (%)	68.17	77.74	69.92	84.75	80.55	**89.02**
Kappa coefficient	0.65	0.76	0.67	0.84	0.79	**0.88**

**Table 4:** Average classification accuracy comparison of the four classifiers.

Method	MLC	SVM	MLC (Without Interference)	SVM (Without Interference)	DD	ISBDD
Water (%)	92.24	95.66	91.72	94.32	93.27	**97.72**
Paddy (%)	69.45	80.00	82.09	98.07	71.74	**99.78**
Caraway (%)	98.37	98.10	**100.00**	99.46	99.52	98.85
Wild-grass (%)	79.58	72.09	92.98	94.56	**96.65**	92.20
Pachyrhizus (%)	88.72	90.05	96.97	**97.91**	96.78	95.26
Tea (%)	95.08	97.13	98.30	98.74	**99.37**	98.73
Bamboo (%)	94.40	96.61	90.52	92.69	94.72	**96.89**
Overall accuracy (%)	85.74	89.20	90.85	96.26	89.46	**97.65**
Kappa coefficient	0.83	0.87	0.89	0.95	0.87	**0.97**
